# Supplementary material for: DFT Studies on the Antioxidant Activity of Naringenin and Its Derivatives: Effects of the Substituents at C3
Source: Int J Mol Sci. 2019 Mar 22;20(6):1450. doi: 10.3390/ijms20061450 (PMC6470621; doi:10.3390/ijms20061450)
Supplement: Supplementary file 1 [file ijms-20-01450-s001.pdf]

Supplementary materials for:

DFT studies on the antioxidant activity of naringenin and its derivatives: effects of the substituents at C3

Yan-Zhen Zheng [1], Geng Deng [2], Rui Guo [1], Da-Fu Chen [1,*] and Zhong-Min Fu [1]

[1] College of Bee Science, Fujian Agriculture and Forestry University, Fuzhou 350002, P. R. China; yanzhenzheng@fafu.edu.cn (Y.Z.Z.); rui_0508@163.com (R.G.); dfchen826@fafu.edu.cn (D.-F.C.); 369699776@qq.com (Z.-M.F)

[2] Key Laboratory of Bioorganic Phosphorous Chemistry and Chemical Biology (Ministry of Education), Department of Chemistry, Tsinghua University, Beijing 100084, P. R. China; dengg@mail.tsinghua.edu.cn (G.D.)

* Correspondence: dfchen826@fafu.edu.cn (D.F.C.); Tel.: +86-0591-8378-9482

Table S1. ΔBDE in kJ/mol obtained by the difference between BDE of the substituted naringenin and naringenin itself.

	Gas			Benzene			Water		
	4′–OH	5–OH	7–OH	4′–OH	5–OH	7–OH	4′–OH	5–OH	7–OH
NH_2	-2.3	-2.7	-1.4	-1.9	-2.2	-1.6	-1.5	-1.3	-2.4
OH	-1.1	-1.9	-1.0	-0.7	-1.7	-1.4	-0.7	-1.1	-1.8
OMe	-1.3	-2.2	-0.7	-0.9	-1.9	-1.0	0.2	-1.0	-1.3
Me	0.5	-0.8	-0.2	0.6	-0.5	-0.7	-0.1	-0.6	-0.9
F	2.1	1.1	0.4	2.2	0.6	0.9	2.4	0.4	1.4
Cl	2.8	2.1	2.8	3.4	0.7	1.4	4.5	0.9	2.1
CHO	3.4	2.9	3.2	3.7	0.9	3.2	4.8	1.5	2.6
CF_3	4.5	3.2	3.7	4.0	1.5	4.1	4.9	2.4	3.5
CN	5.6	3.9	5.1	5.7	2.3	5.7	5.1	2.7	5.1
NO_2	7.2	4.4	6.2	6.9	2.9	7.1	6.2	3.1	7.1

Table S2. The Hammett sigma constants (σ_m and σ_p)[a], the field/inductive parameter (*F*)[a] and resonance parameter (*R*)[a] of the substituents.

	σ_m	σ_p	*F*	*R*
H	0.00	0.00	0.00	0.00
NH_2	-0.16	-0.66	0.08	-0.74
OH	0.12	-0.37	0.33	-0.70
OMe	0.12	-0.27	0.29	-0.56
Me	-0.07	-0.17	0.01	-0.18
F	0.34	0.06	0.45	-0.39
Cl	0.37	0.23	0.42	-0.19
CHO	0.35	0.42	0.33	0.09
CF_3	0.43	0.54	0.38	0.16
CN	0.56	0.66	0.51	0.15
NO_2	0.71	0.78	0.65	0.13

[a] Data from work by Hansch, Leo, and Taft (1991).

Table S3. ΔIP in kJ/mol obtained by the difference between IP of the substituted naringenin and naringenin itself.

	Gas	Benzene	Water
NH_2	-4.1	-3.6	-6
OH	6.8	1.4	1
OMe	4.7	2.6	-1.9
Me	-3.3	-1.4	-1
F	22.7	18.9	8.9
Cl	18.3	17.4	9.8
CHO	19.4	18	12.6
CF_3	22.8	19.8	11.1
CN	35.5	28.9	12.8
NO_2	41.4	35.4	18.8

Table S4. ΔPA in kJ/mol obtained by the difference between PA of the substituted naringenin and naringenin itself.

	Gas			Benzene			Water		
	4′–OH	5–OH	7–OH	4′–OH	5–OH	7–OH	4′–OH	5–OH	7–OH
NH_2	3.2	2.4	5.5	3.0	3.2	2.4	1.9	1.7	2.6
OH	-0.6	-2.7	-10.2	-1.3	-4.7	-3.4	-0.3	-6.4	-0.5
OMe	-0.8	-9.9	-7.5	-1.4	-6.5	-4.4	-0.4	-2.7	-0.8
Me	0.6	2.0	2.9	1.2	2.4	0.8	0.3	1.4	0.4
F	-2.3	-21.7	-18.3	-1.4	-18.7	-14.3	-0.8	-10.2	-5.7
Cl	-1.7	-21.3	-18.4	-1.8	-17.6	-14.2	-1.1	-8.1	-5.6
CHO	-3.4	-26.4	-22.3	-2.8	-18.8	-14.6	-0.8	-7.5	-4.1
CF_3	-5.2	-26.3	-21.0	-3.0	-20.3	-15.1	-2.1	-8.4	-5.0
CN	-6.7	-38.3	-32.7	-5.6	-29.4	-23.1	-3.0	-11.3	-7.1
NO_2	-8.5	-45.7	-38.4	-7.4	-35.8	-27.8	-3.7	-18.0	-10.7
